# Supplementary material for: Functionalization of Octacalcium Phosphate Bone Graft with Cisplatin and Zoledronic Acid: Physicochemical and Bioactive Properties
Source: Int J Mol Sci. 2023 Jul 19;24(14):11633. doi: 10.3390/ijms241411633 (PMC10380611; doi:10.3390/ijms241411633)
Supplement: Supplementary file 1 [file ijms-24-11633-s001.zip › ijms-2422892-supplementary.pdf]

SUPPLEMENTARY MATERIAL

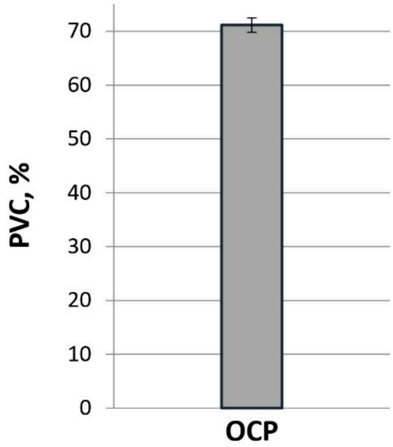

Figure S1 – Data of observation of OCP ceramics in indirect contact with MG-63 cells (24 hours): MTT assay, PVC in comparison with control.

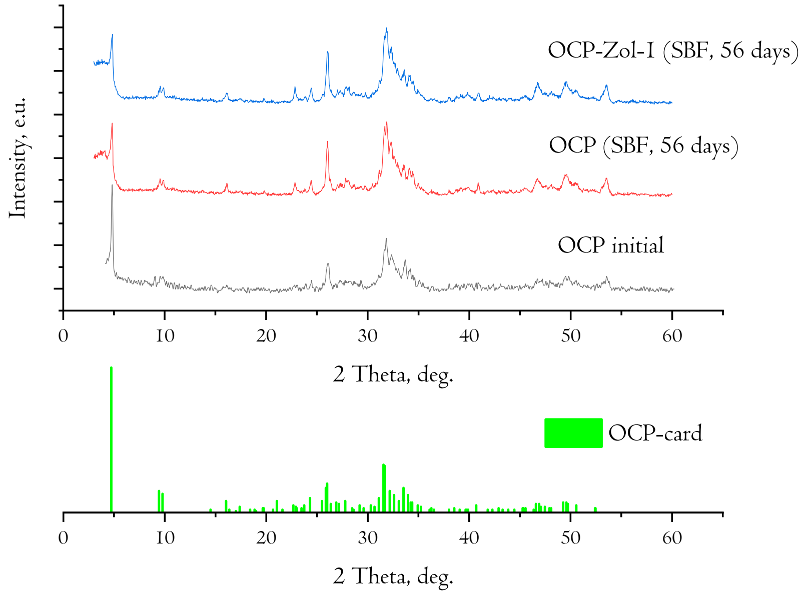

Figure S2 – XRD patterns of drug-free and functionalized with Zol OCP ceramics after exposure to SBF during 56 days. As SEM study revealed the appearance of rounded particles on OCP surface when it was functionalized with Zol and exposure to SBF (Figure 11B, C), it was obvious to reveal it, using XRD. However, the distribution of these particles on the surface of OCP ceramics was unregular and counted an insignificant part of the total material volume, therefore it was irrelevant to perform XRD indexation of this phase. With this the hydrolysis of OCP to HA took place during exposure to SBF that confirmed by decreasing of the main diffraction peak at $2\theta=4.7$ deg.

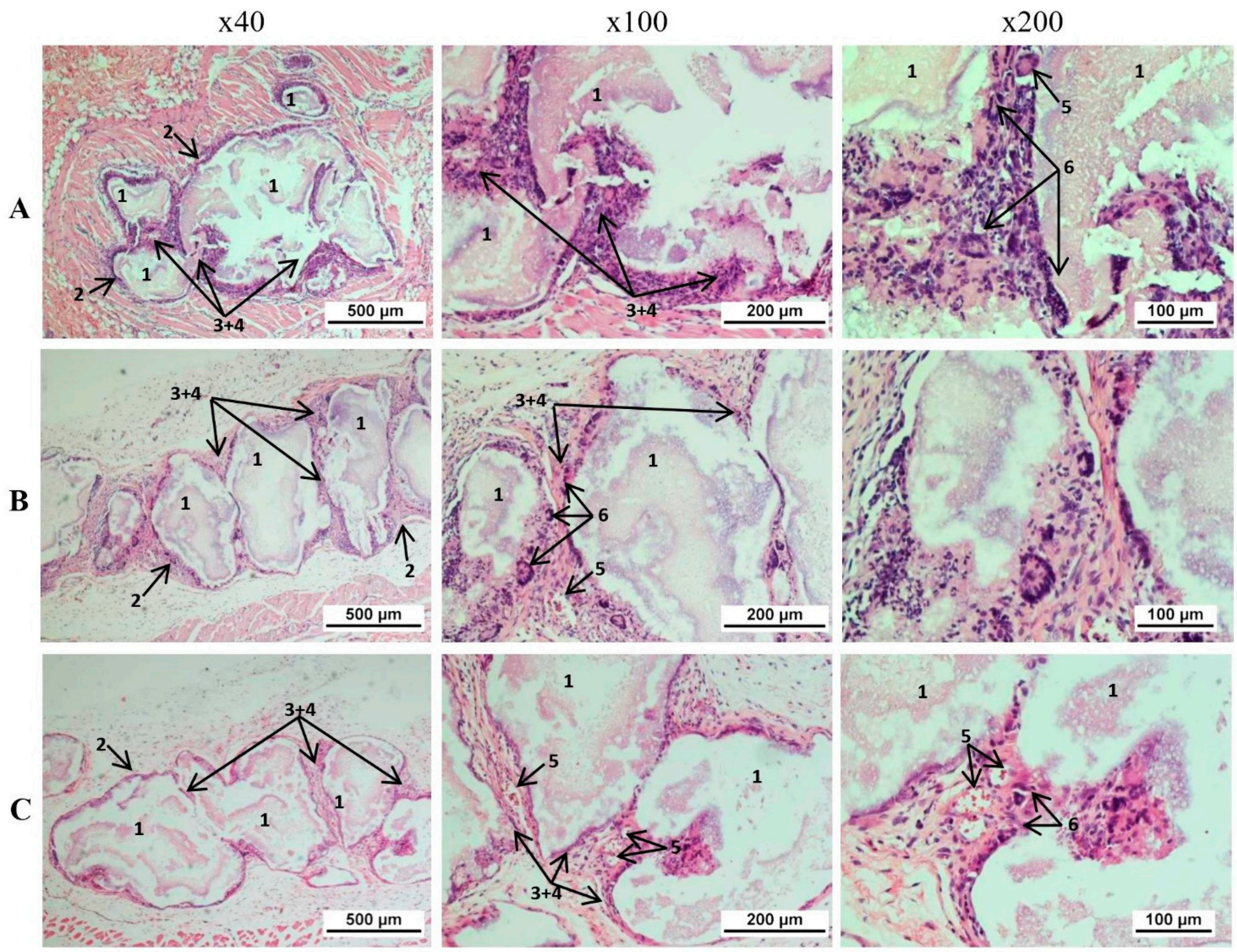

Figure S3 (15) – Histological analysis of subcutaneous tissue with implanted OCP ceramics at different time points: A – 4 weeks, B – 8 weeks, C – 12 weeks; hematoxylin-eosin staining: 1 – OCP granule; 2 – fibrous capsule; 3 – intergranular connective tissue; 4 – lymphocytic infiltration;5 – blood vessel; 6 – foreign-body giant cell.

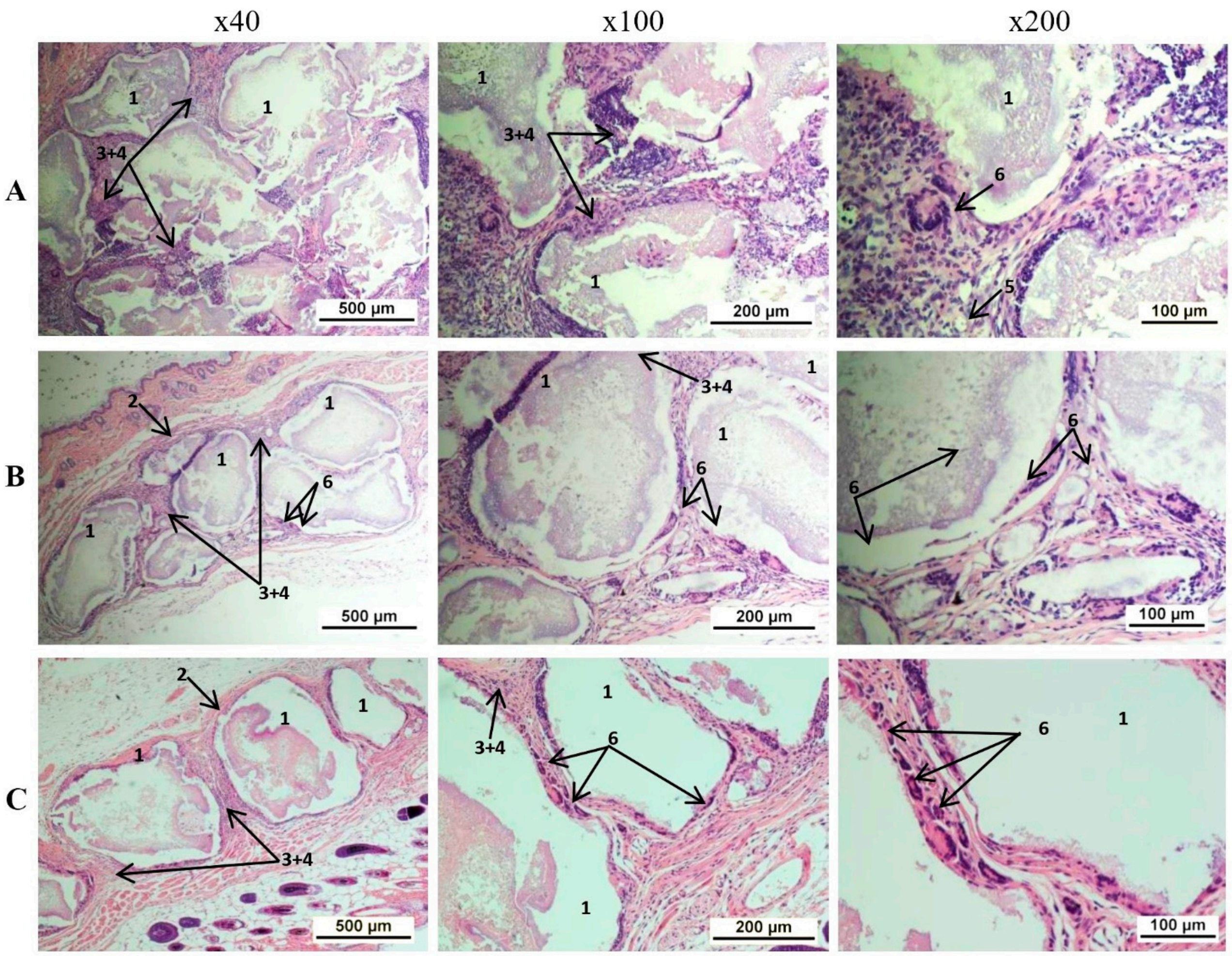

Figure S4 (16) – Histological analysis of subcutaneous tissue with implanted OCP-Cis ceramics atdifferent time points: A – 4 weeks, B – 8 weeks, C – 12 weeks; hematoxylin-eosin staining: 1 – OCP granule; 2 – fibrous capsule; 3 – intergranular connective tissue; 4 – lymphocytic infiltration;5 – blood vessel; 6 – foreign-body giant cell.

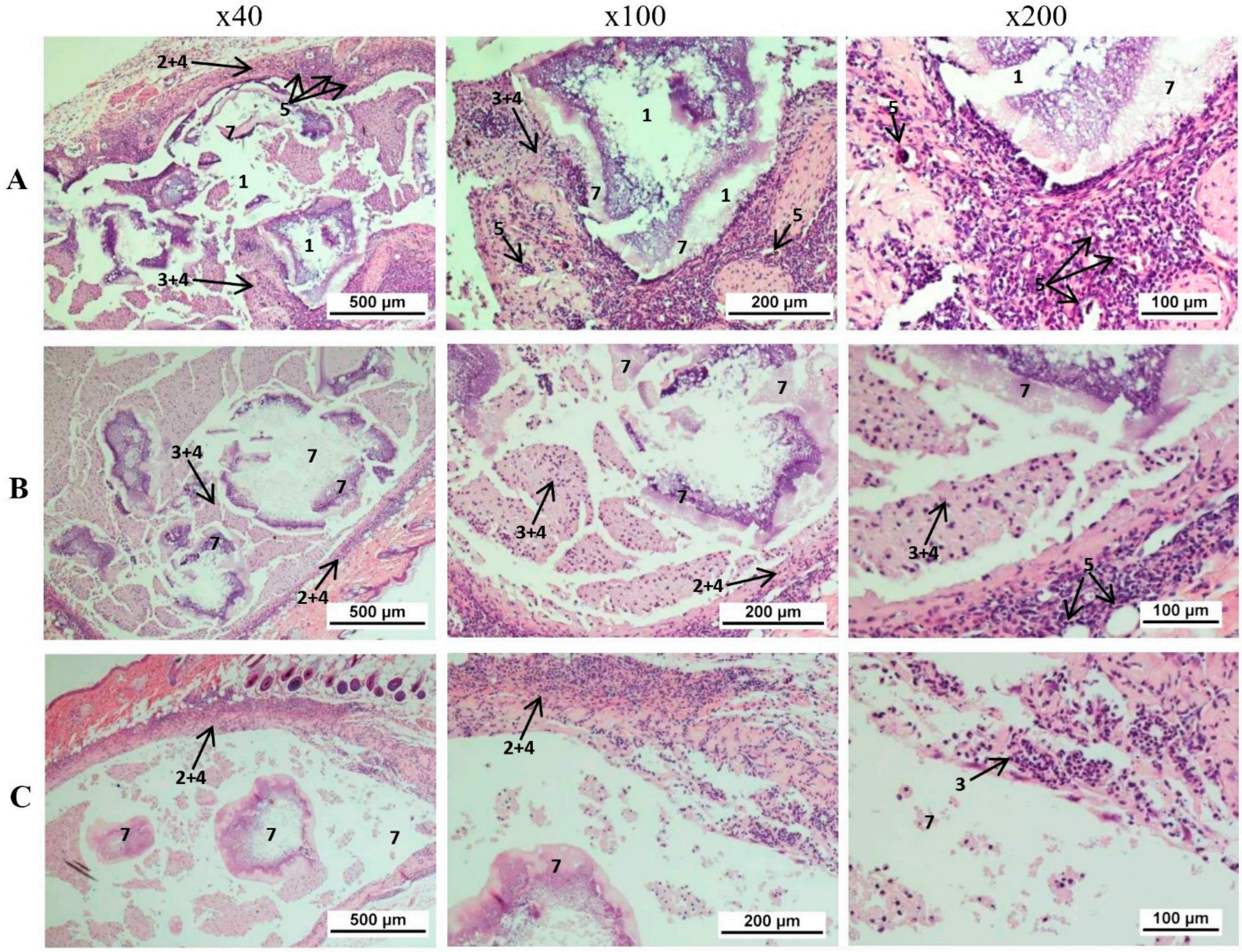

Figure S5 (17) – Histological analysis of subcutaneous tissue with implanted OCP-Zol ceramics atdifferent time points: A – 4 weeks, B – 8 weeks, C – 12 weeks; hematoxylin-eosin staining: 1 – OCP granule; 2 – fibrous capsule; 3 – intergranular connective tissue; 4 – lymphocytic infiltration;5 – blood vessel; 6 – foreign-body giant cell; 7 – amorphous colloidal substance.

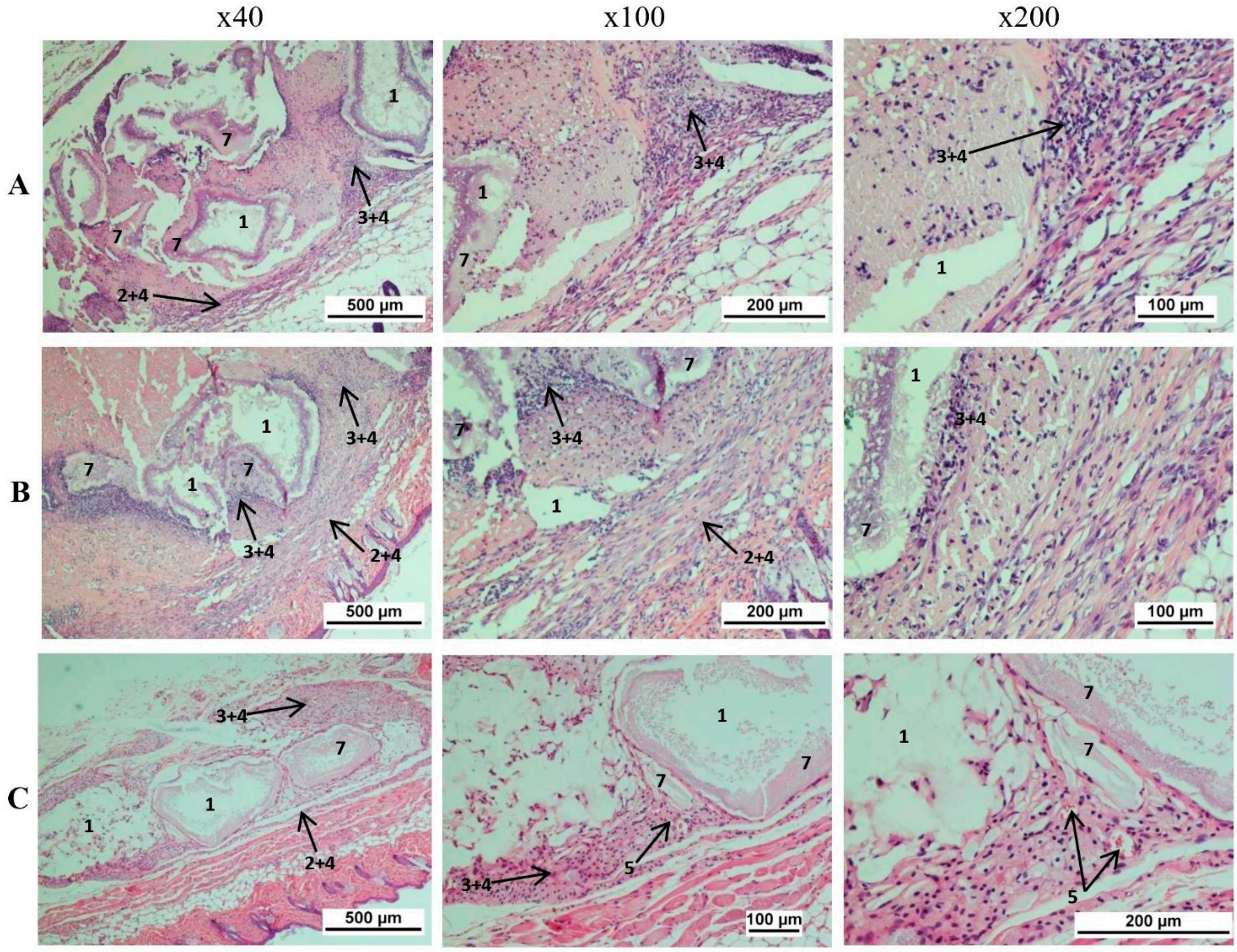

Figure S6 (18) – Histological analysis of subcutaneous tissue with implanted OCP-Cis/Zol ceramicsat different time points: A – 4 weeks, B – 8 weeks, C – 12 weeks; hematoxylin-eosin staining: 1 – OCP granule; 2 – fibrous capsule; 3 – intergranular connective tissue; 4 – lymphocytic infiltration; 5 – blood vessel; 6 – foreign-body giant cell; 7 – amorphous colloidal substance.

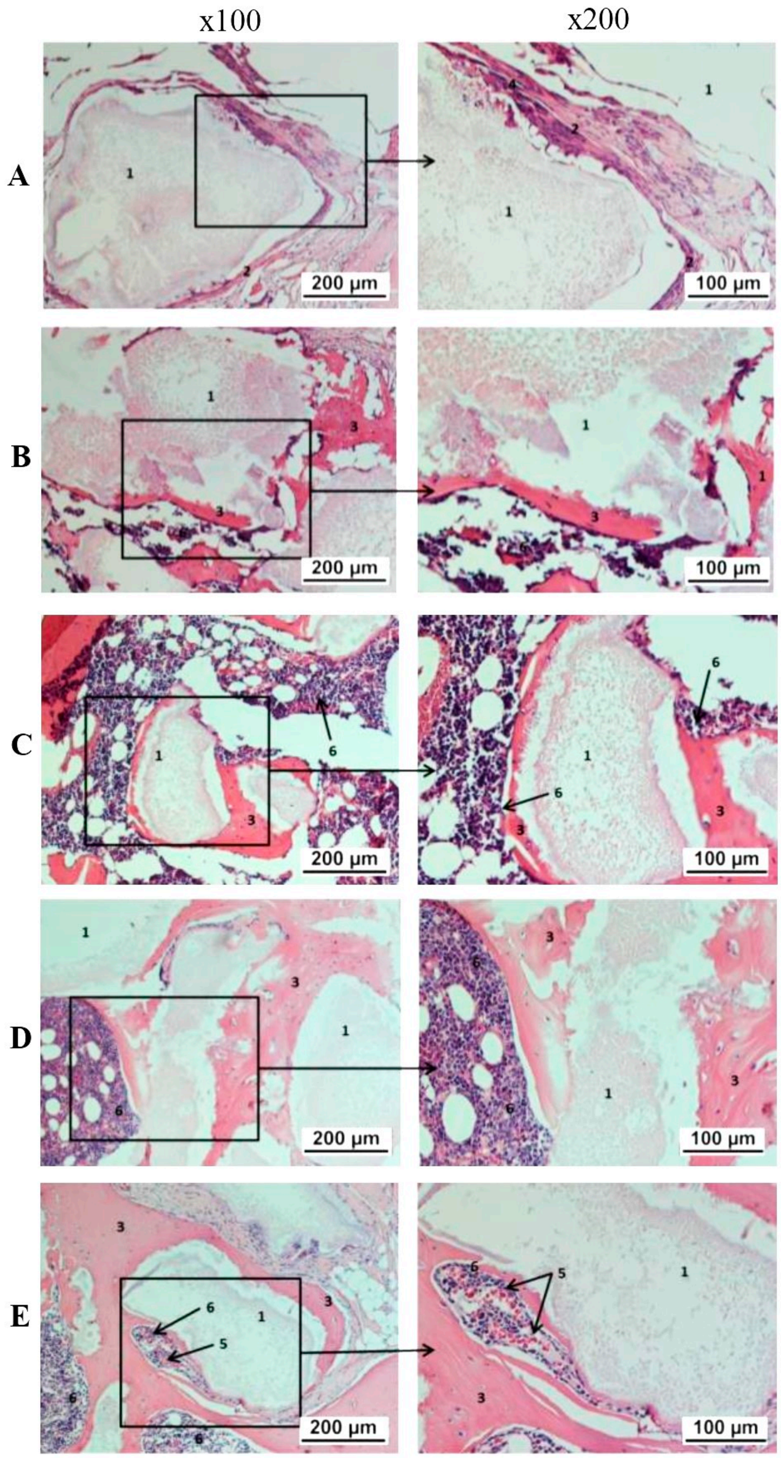

Figure S7 (19) – Histological analysis of rat tibia defect with implanted OCP ceramics at different time points: A, B – 4 weeks, C – 8 weeks, D – 12 weeks, E – 6 months; hematoxylin-eosin staining: 1 – OCP granule, 2 – connective tissue, 3 – bone tissue, 4 – foreign-body giant cells, 5 – blood vessel, 6 – bone marrow hematopoiesis.

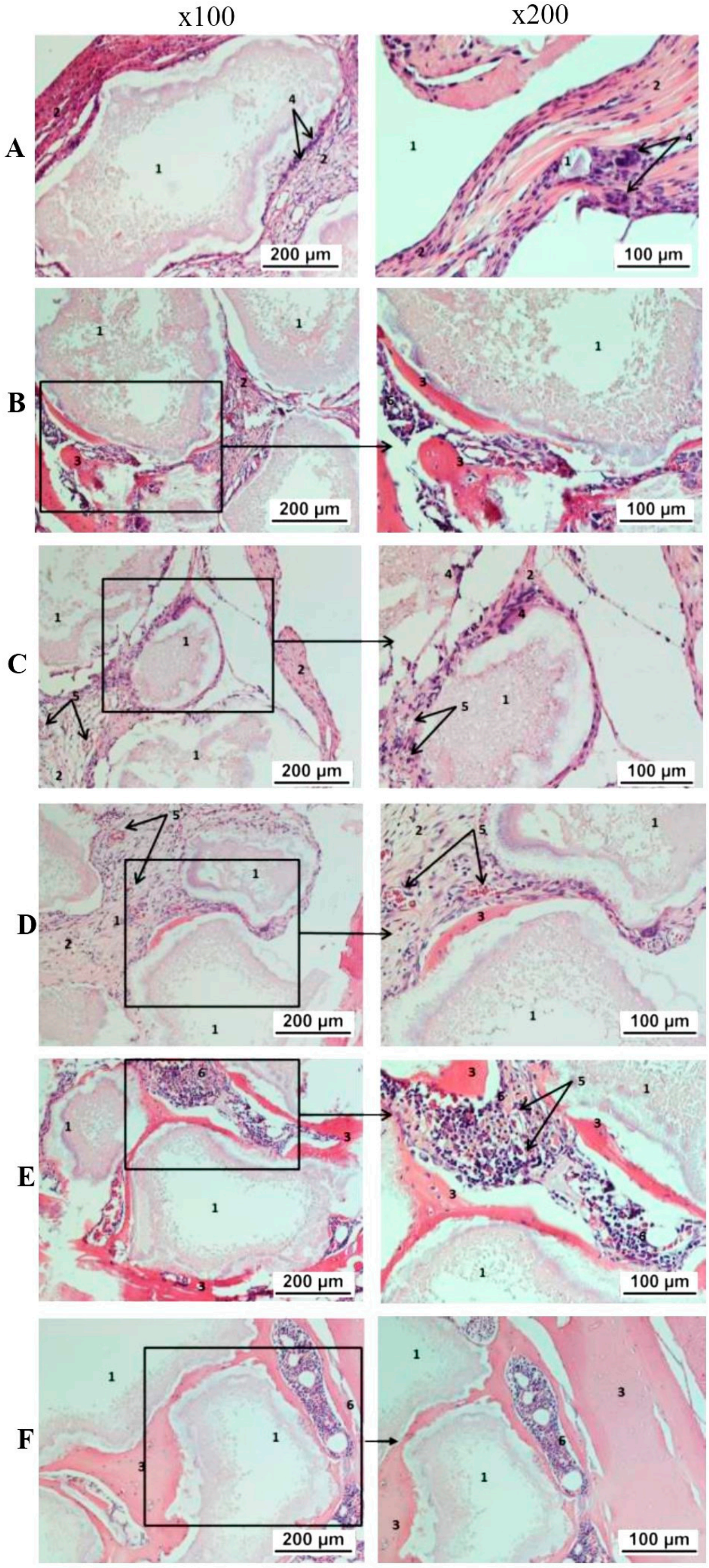

Figure S8 (20) – Histological analysis of rat tibia defect with implanted OCP-Cis ceramics at different time points: A – 4 weeks, B, C – 8 weeks, D, E – 12 weeks, F – 6 months; hematoxylin- eosin staining: 1 – OCP granule, 2 – connective tissue, 3 – bone tissue, 4 – foreign-body giant cells,5 – blood vessel, 6 – bone marrow hematopoiesis.

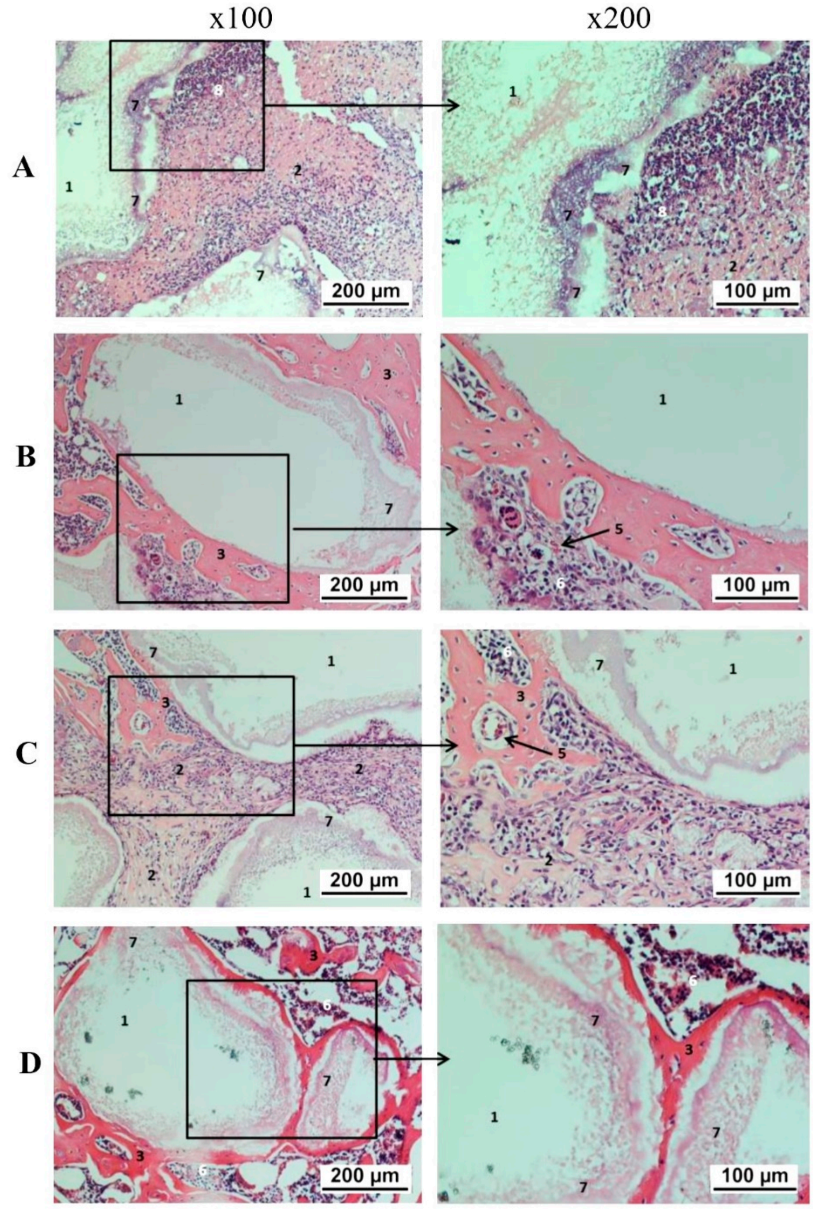

Figure S9 (21) – Histological analysis of rat tibia defect with implanted OCP-Zol ceramics at differenttime points: A – 4 weeks, B, C – 8 weeks, D – 12 weeks; hematoxylin-eosin staining: 1 – OCP granule, 2 – connective tissue, 3 – bone tissue, 4 – foreign-body giant cells, 5 – blood vessel, 6 – bonemarrow hematopoiesis.

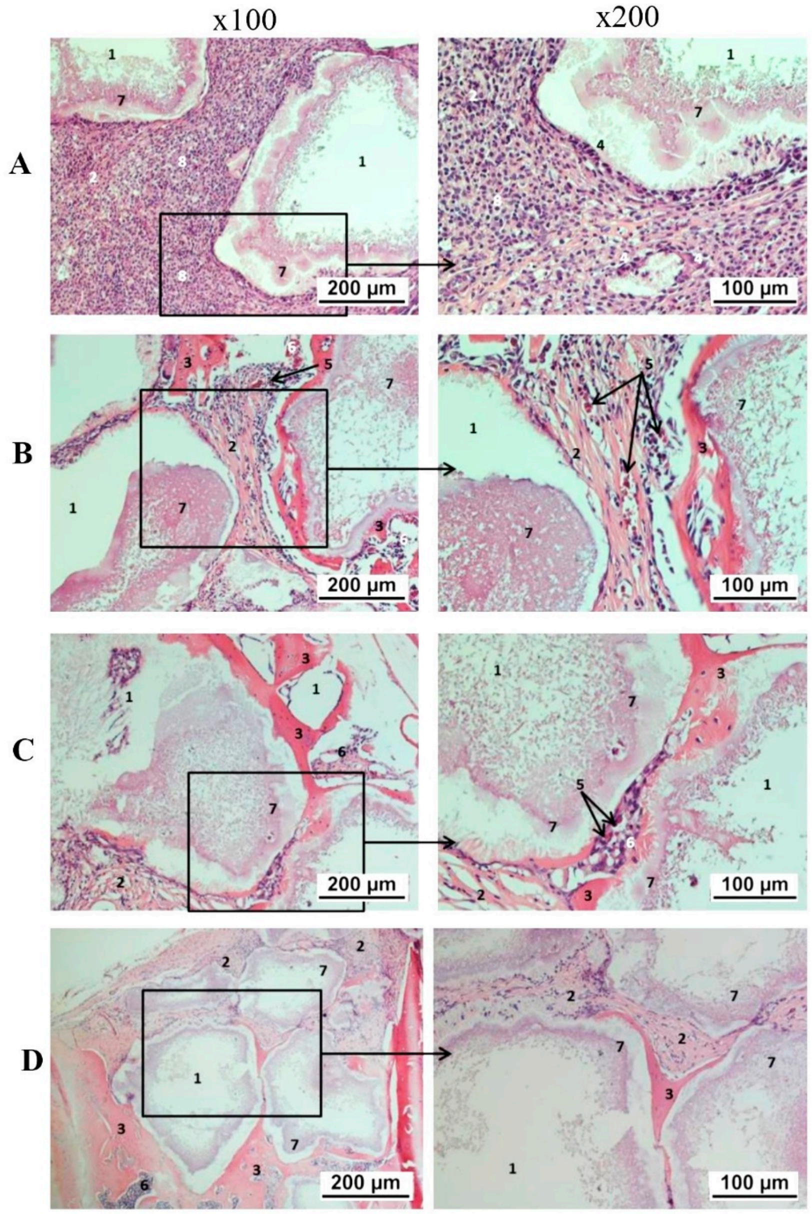

Figure S10 (22) – Histological analysis of rat tibia defect with implanted OCP-Cis/Zol ceramics at different time points: A – 4 weeks, B – 8 weeks, C– 12 weeks, D – 6 months; hematoxylin-eosin staining: 1 – OCP granule, 2 – connective tissue, 3 – bone tissue, 4 – foreign-body giant cells, 5 – blood vessel, 6 – bone marrow hematopoiesis, 7 – amorphous colloidal substance, 8 – lymphocyticinfiltration.

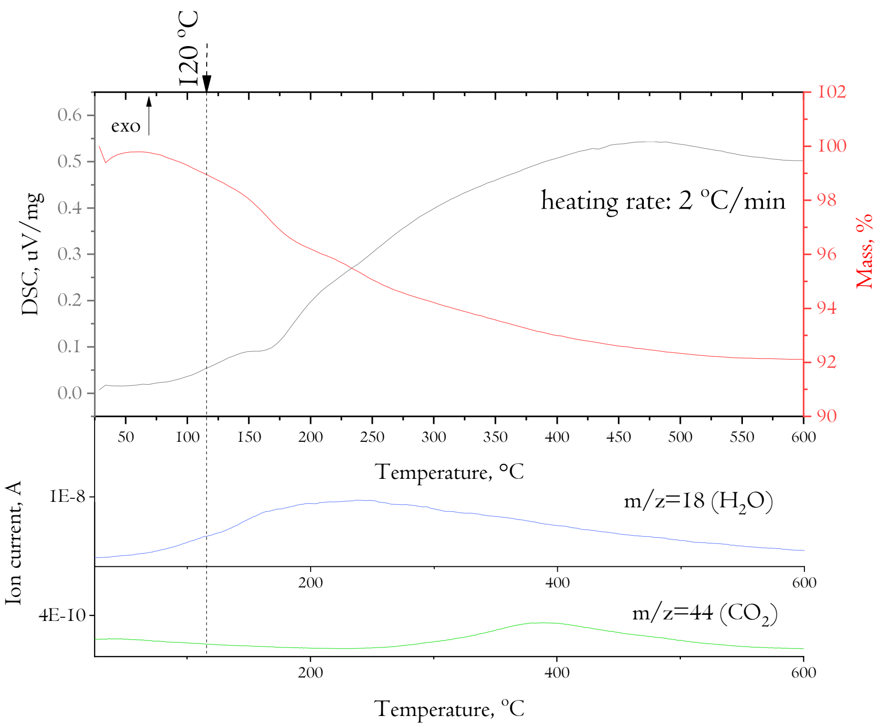

Figure S11 – Results of DSC analyses of OCP treated to 600 °C with a heating rate of 2.5 deg./min in the way of revealing of the influence of sterilization conditions on OCP structure changes. Mass loss starting from a temperature of about 60-70 °C, corresponds to a loss of adsorbed water. This is confirmed by mass spectra (m/z=18). The greatest mass loss is observed in the range above 150 °C. Mass loss is also accompanied by the removal of carbonate groups remaining after the transformation procedure of DCPD to OCP in acetate solution, and also adsorbed from the air (m/z=44) [100]. So, during heating, first adsorbed water is removed, then the structural water – with partial hydrolysis of OCP to HA. At 120 °C, the hydrolysis is insignificant, and perhaps even preferable – for the absence of abrupt processes accompanied by the release of orthophosphate groups, leading to acidification of the surface, and with a slight initial hydrolysis, this acidification will not lead to undesirable cytotoxic reactions during *in vitro* studies.

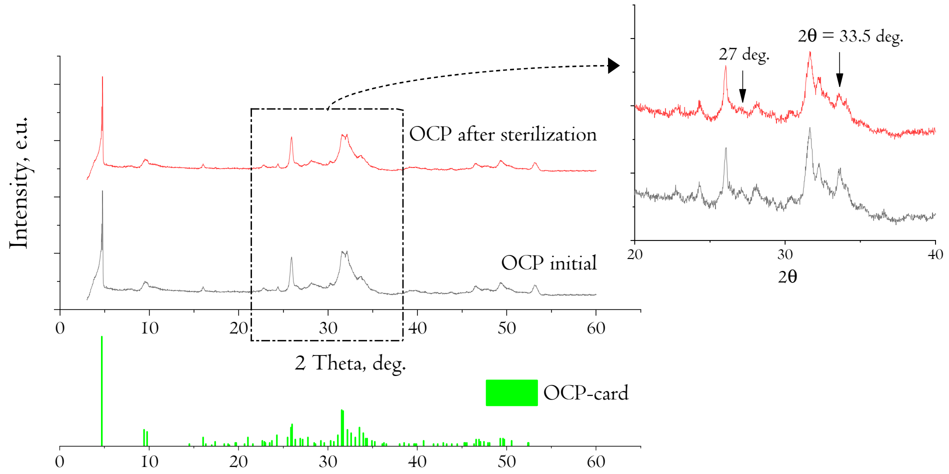

Figure S12 – XRD patterns of OCP before and after sterilization. Demonstration of the correlation of the main peaks to OCP phase. Some degradation of OCP after sterilization is noticeable, in accordance to a decrease in the intensity of peaks of 2θ=27 and 33.5 deg. That confirms a slight OCP conversion to HA, but quite insignificant, since the ratio of the main peaks remains. The ratio of the main peaks changes significantly for the materials in this study that have been exposure to SBF for 56 days (Fig. S2).

Table S1. Gene symbols and sequences of primers of the analyzed and normalized genes

№	Gene symbol	Encoded protein	F and R primer sequences 5'-3'
1	*RUNX2*	Runt-associated transcriptionfactor 2 is one of the key regulators of osteoblastic cells (human)	F: tca-acg-atc-tga-gat-ttg-tgg-g<br>R: ggg-gag-gat-ttg-tga-aga-cgg
2	*SP7*	Osterix is a transcription factor involved in the differentiation of mesenchymal progenitors into osteoblasts and osteocytes (human)	F: ccc-acc-tac-cca-tct-gac-tt<br>R: gct-gcc-cac-tat-ttc-cca-ct
3	*ALPL*	Alkaline phosphatase is a membrane-bound glycosylatedenzyme involved in matrix mineralization (human)	F: acc-acc-acg-aga-gtg-aac-ca<br>R: cgt-tgt-ctg-agt-acc-agt-ccc
4	*GAPDH-1*	Glyceraldehyde-3-phosphate dehydrogenase, housekeepinggene (human)	F: gaa-ggt-gaa-ggt-cgg-agt-c<br>R: gaa-gat-ggt-gat-ggg-att-tc
5	*TRAP*	Tartrate Resistant acid phosphatase (mice)	F: cac-tcc-cac-cct-gag-att-tgt<br>R: ccc-cag-aga-cat-gat-gaa-gtc-a
6	*GAPDH-2*	Glyceraldehyde-3-phosphate dehydrogenase, housekeepinggene (mice)	F: agg-tcg-gtg-tga-acg-gat-ttg<br>R: tgt-aga-cca-tgt-agt-tga-ggt-ca
